# Generation and Application of Ultra-Fine, Long-Term Stable Nanobubble Water: An Evaluation of Inclusion Effects on Aromatic Components and Antimicrobial Activity

**DOI:** 10.3390/ijms27156909

**Published:** 2026-08-01

**Authors:** Shin Shimizu, Mikiko Tanaka, Nanami Tominaga, Katsuyuki Fujinami, Keita Takanashi, Katsuaki Dan

**Affiliations:** 1 Symbiosis Inc., Osaka 534-0025, Japan; 2 Intestinal Microbiota Transplantation Clinical Research Inc., Osaka 534-0025, Japan; 3 FSX Inc., Tokyo 186-0012, Japan; 4 Department of Pathophysiology, Yokohama University of Pharmacy, Yokohama 245-0066, Japan; keita.takanashi@yok.hamayaku.ac.jp; 5 Division of Research and Development, Research Organization of Biological Activity, Tokyo 150-0021, Japan

**Keywords:** nanobubble water, intestinal flora transplantation (FMT), autism spectrum disorder (ASD), hydrogen, ozone, oxygen, VirusBlock, citral, cyclodextrin

## Abstract

Nano- and pico-bubble water (NPB), containing hydrogen or ozone, is widely used as a cleaning agent due to its bactericidal and antiviral properties. However, some products—such as certain hydrogen waters—contain only large bubbles or have an extremely low bubble count, making them sometimes indistinguishable from ordinary drinking water. To accurately evaluate NPB activity, we developed a method for producing ultra-nano–pico-bubble water (NanoGAS water [NGW]), an ultra-fine bubble water that is stable and non-volatile over extended periods. By combining a mixed gas–liquid fluid rotary mixer and a shear filter, we produced ultra-fine bubbles that could be sealed in water. This method produced bubbles that remained stable in water even after 10 years since production. NGW has been clinically evaluated as a solvent for fecal microbiota transplantation (FMT) and has been demonstrated to be effective at improving bacterial engraftment in the intestinal tract in patients with autism spectrum disorder (ASD). Furthermore, encapsulating specific gases (hydrogen and ozone) can achieve more diverse effects. In this study, we evaluated the aroma-encapsulating effects, as well as the strength and persistence of the antimicrobial activity, of novel NGW formulations (Air-NGW, H_2_-NGW, S-O_3_-NGW, and L-O_3_-NGW). Both H_2_-NGW and O_3_-NGW generated in this study demonstrated slight inclusion activity with volatile aromatic compounds (citral). Furthermore, both H_2_-NGW (at ≥10% dilution) and O_3_-NGW (even at a 1% dilution) exhibited sustained antibacterial efficacy against general viable bacteria for 24 weeks. Moreover, additive effects were observed when combined with antibacterial and antiviral compounds (polyoxometalates) developed by the authors. While further consideration, including cost-effectiveness, is needed to translate these findings into practical applications, they provide a fundamental framework for future research.

## 1. Introduction

Systems utilizing micro–nano-bubbles are being applied to technological innovations in fields such as environmental conservation—including wastewater treatment [1,2,3,4,5,6,7,8,9]—as well as basic medicine [10,11,12,13,14,15,16,17,18,19,20,21,22,23,24,25,26,27,28,29,30], agriculture [31,32,33,34,35,36,37,38,39,40,41,42,43,44], and fisheries [45,46,47]. Nano–pico-bubble water, such as hydrogen water and ozone water, is employed in various cleaning solutions owing to its bactericidal and antiviral effects. Hydrogen water is also widely consumed as drinking water due to its potential antioxidant properties [10,11,12,13]. However, no clear standards have been established for commercially available hydrogen water and similar products, with some products containing large bubbles or a low initial number of bubbles. This results in rapid hydrogen dissipation, rendering these products the same as regular drinking water. Thus, insufficient product development and exaggerated, unsubstantiated marketing claims undermine consumer trust and negatively impact our perceptions of the broader health industry. Hydrogen water has been demonstrated to significantly reduce lipid peroxide levels in the rat liver, thus mitigating oxidative stress [12], attenuating inflammation by intestinal bacteria [13], and exerting neuroprotective effects [14]. Moreover, ozone water has been demonstrated to exhibit antibacterial and antiviral effects (against *Escherichia coli* O157:H7 and *Salmonella* species) due to its oxidizing capacity [15,16,17]. Although several clinical trials have been conducted, their findings are inconsistent, and conclusive evidence has not yet been obtained [18]. It has also become clear that stable micro–nano-bubbles are difficult to generate and that there is a strong correlation between bubble size and duration [2,48]. Therefore, it is essential to develop ultra-nano–pico-bubble water (hereafter referred to as NanoGAS water; NGW) characterized by ultra-fine nano- and pico-sized bubbles with long-term stability and evaluate its biological activity and potential applications. Traditionally, ultra-fine-bubble generators are used to physically shear bubbles using rotating blades. However, this method is inefficient for generating ultra-fine bubbles. To overcome this, we successfully combined a mixed gas–liquid fluid rotary mixer and a shear filter to generate nano-sized or smaller bubbles. 

Disruptions in the gut microbiota (dysbiosis) are increasingly linked to various diseases, including gastrointestinal, metabolic, autoimmune, and psychiatric disorders [49,50], with fecal microbiota transplantation (FMT) attracting attention as a therapeutic strategy for improving dysbiosis. Studies in 1958 [51] and 2013 [52] demonstrated that FMT resulted in significantly higher relapse-free cure rates for recurrent *Clostridium difficile* infection (rCDI) compared to conventional oral vancomycin treatment. Accordingly, the Infectious Diseases Society of America (IDSA) guidelines strongly recommend FMT for rCDI [53]. Moreover, novel treatment protocols for ulcerative colitis (UC) are being explored, including a combination of allergic antibiotic therapy (AFM) and FMT (antibiotic-FMT; A-FMT) [54,55].

Using NGW as a bacterial suspension medium in FMT enhances the engraftment efficiency of bacteria transplanted into the recipient gut, demonstrating efficacy and safety in clinical trials in autism spectrum disorder (ASD) patients [56]. Studies in a diabetic mouse model indicate that NGW may lower blood glucose levels through interactions between gut bacteria and intestinal epithelial cells, alongside inhibition or delay of glucose uptake [57].

The bubbles contained in this NGW can contain various gases, including air, hydrogen, ozone, and oxygen. Furthermore, by modulating the introduction pressure and time of the displacement gas, NGW with varying bubble sizes can be generated.

Preliminary studies (unpublished internal company documents) demonstrated that H_2_-NGW can prevent rust on aluminum materials for four years (Supplement Figure S1); inhibit the oxidation of alcoholic beverages (Supplement Figure S2); enhance the extraction efficiency of glutamic acid, an umami component, using kelp broth, resulting in faster broth production (Supplement Figure S3); and increase the fermentation efficiency of koji by over 40% (Supplement Figure S4).

Other research has reported that H_2_-NGW suspensions of *E. coli* and *Staphylococcus aureus* exhibit significantly lower bacterial counts within 6 h or 3 h, respectively, compared to physiological saline or sterile water for injection [58]. Furthermore, this formulation reduced oxidative stress markers in adipose tissue [59].

This study presents new findings obtained through cross-industry collaboration among companies developing medical-grade ultra-fine bubble water [56,57,60], developers of antibacterial and antiviral compounds [57,61,62,63,64,65,66], and manufacturers incorporating these compounds and fragrances into commercial hand towels [62,63,64,65,66]. Additionally, our group has investigated the biological activity of polyoxometalates (PMs) for several years [61]. By combining two antimicrobial and antiviral PMs (Keging-type PMs) selected from several PMs with antitumor, antibacterial, and antiviral activity, we developed the broad-spectrum antiviral formulation VirusBlock (VB^®^). This formulation has been incorporated in cosmetic-grade antimicrobial and antiviral wet wipes, which have increased in demand, particularly since the COVID-19 pandemic [62,63,64,65,66].

Typically, wet wipes are classified as general consumer goods with limited use for hand hygiene, whereas cosmetic-grade products can be used on the entire epidermis.

Incorporating VB^®^ as a cosmetic ingredient has increased its versatility. Moreover, VB^®^ possesses antioxidant properties, thus prompting research on its anti-aging effects on skin cells [64,65,66].

Notably, the capacity to stabilize aromatic components (such as 3,7-dimethyl-2,6-octadienal, or citral) in aroma towels for prolonged periods and extend their shelf life would be advantageous from a distribution and economic perspective.

While evaluating antibacterial effects against pathogenic microorganisms is important, when assessing these effects in daily necessities, such as towels and food products, it is equally vital to ensure that general microbial populations are maintained below a certain level threshold. Moreover, the long-term stability of the antimicrobial effect is a key factor for product development.

Based on these considerations, we aimed to evaluate the long-term retention capacity of aroma components in newly developed ultra-fine bubble waters (Air-NGW, H_2_-NGW, and two types of ozonated water with different bubble sizes: S-O_3_-NGW and L-O_3_-NGW) produced using a novel method. First, the effect of including citral in these NGWs was assessed via GC/MS. Subsequently, we assessed the long-term antimicrobial effects of these NGW formulations on total viable bacteria and examined whether they exert additive antibacterial effects when combined with PM.

## 2. Results

### 2.1. Generation of Ultra-Nano–Pico-Bubble Water (NanoGAS Water/NGW)

**Figure 1:**
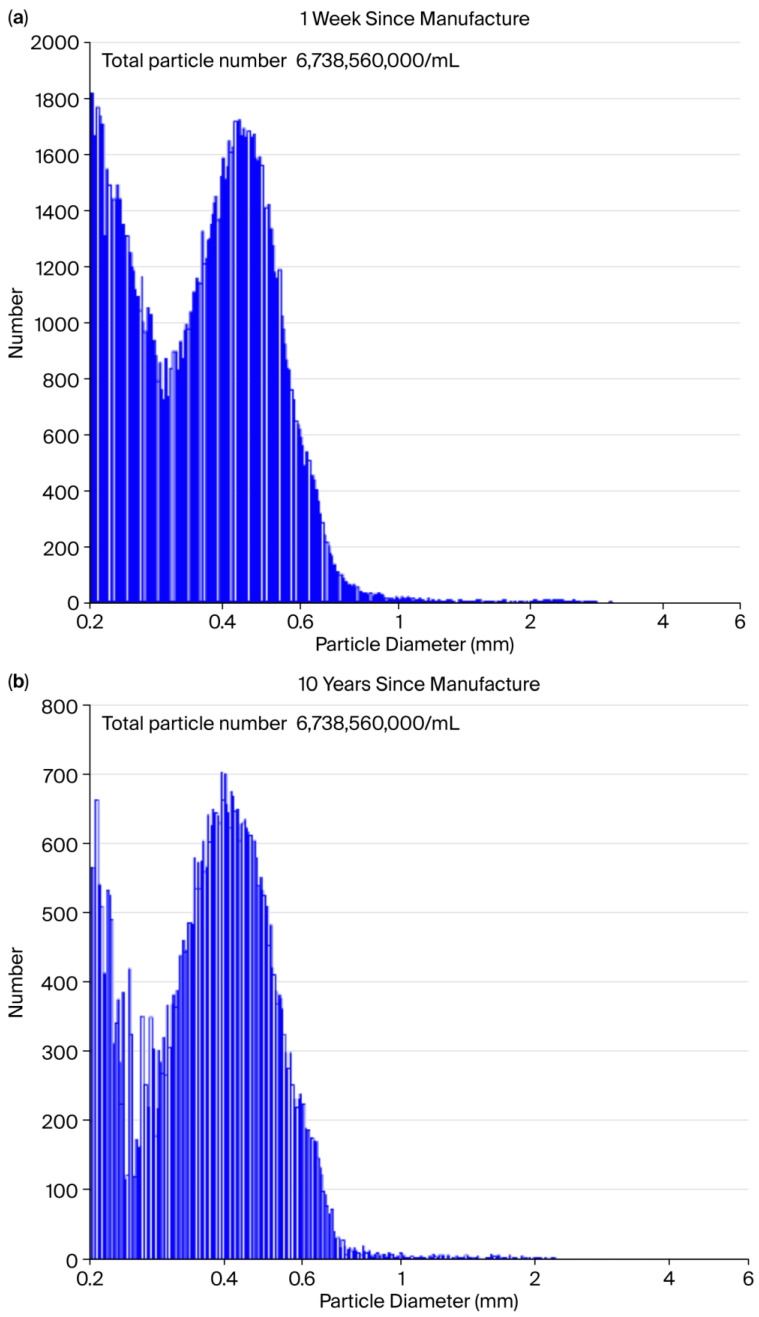
(**a**,**b**). Particle count measurement of ultra-nano–pico-bubble water (NanoGAS water/NGW) using a Coulter counter: (**a**) 1 week since manufacture and (**b**) 10 years since manufacture.

The particle counts of NGW one week and 10 years after generation were approximately 6.7 billion/mL and 2.3 billion/mL, respectively (Figure 1).

### 2.2. Citral Inclusion Capacity of NGW

**Figure 2:**
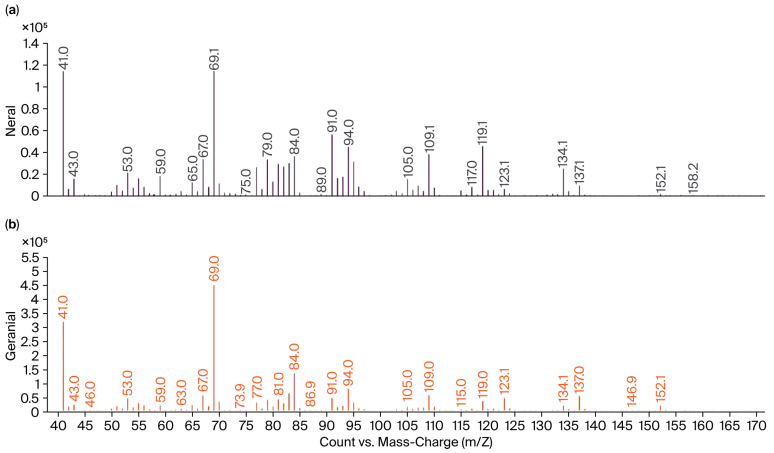
Mass spectra of citral isomers ((**a**): neral and (**b**): geranial).

**Figure 3:**
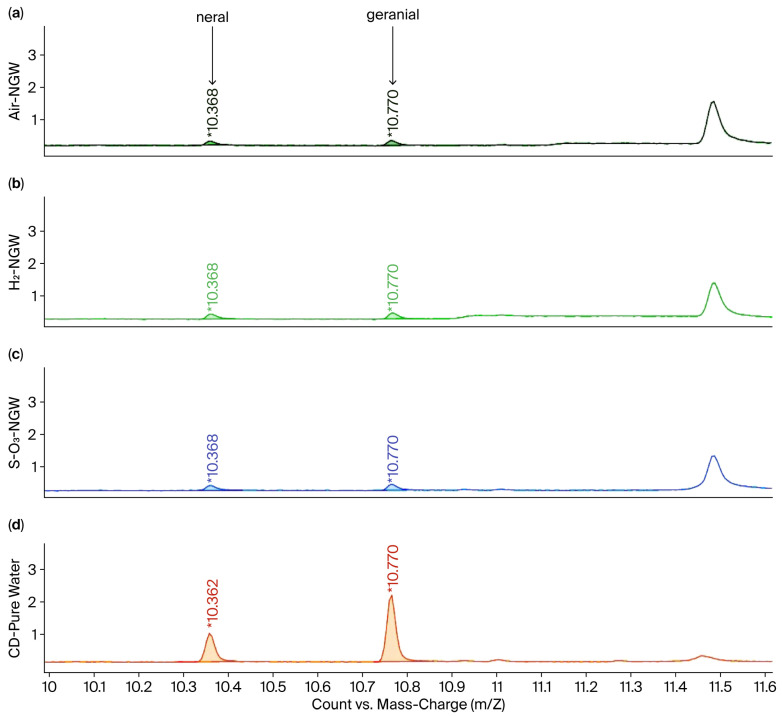
Total ion chromatogram (TIC) of citral isomers (neral and geranial): (**a**) Air-NGW, (**b**) H_2_-NGW, (**c**) S-O_3_-NGW, and (**d**) CD-Pure Water.

**Figure 4:**
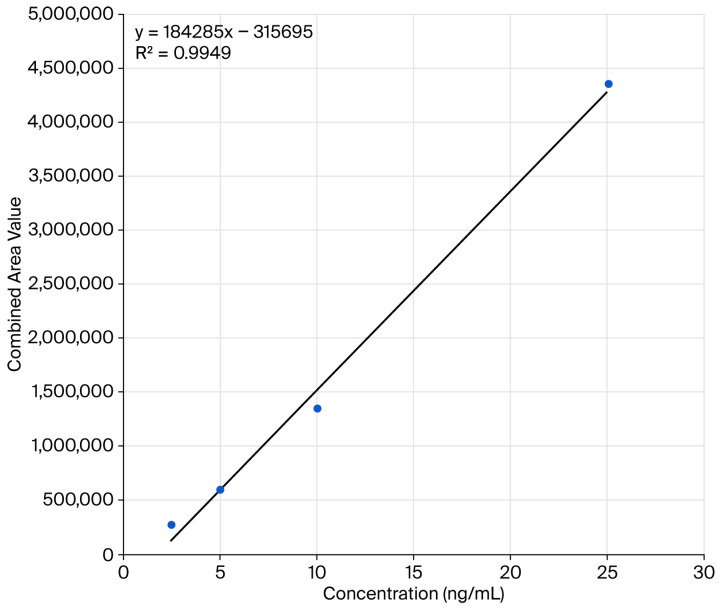
Standard curve of citral based on GC/MS.

The inclusion efficiency of citral in three types of NGW and cyclodextrin (CD; positive control) was quantified using GC/MS, with the GC/MS optimization conditions and calibration curves presented in Figure 2, Figure 3 and Figure 4. The two isomers of citral (neral and geranial) exhibited common peaks in their mass spectra (Figure 2). Furthermore, total ion chromatography (TIC) showed distinct retention times of 10.35–10.37 min for neral and 10.7–10.8 min for geranial, enabling independent detection without interference (Figure 3). The total citral quantity was calculated as the sum of the peak areas for both isomers, and the calibration curve for citral exhibited good linearity, with a correlation coefficient of R^2^ = 0.9949 (Figure 4). Measurements performed two weeks after inclusion revealed citral concentrations of 0.67 mg/mL for Air-NGW, 0.83 mg/mL for H_2_-NGW, 0.92 mg/mL for S-O_3_, and 4.21 mg/mL for CD. Although the inclusion efficiency was lower than that of the positive control (CD; 84.2%), ozone-based formulations exhibited greater inclusion capacity than air- and hydrogen-based formulations (Table 1a).

Therefore, we subsequently investigated whether this effect varied depending on the size of the ozone bubbles. Because the measurements were performed four weeks after inclusion, overall citral concentrations were low, with S-O_3_ and L-O_3_ exhibiting comparable values. Furthermore, increasing the initial amount of citral loaded (sample #3 in each group) resulted in increased inclusion amounts, indicating that there was still additional inclusion capacity (Table 1b).

### 2.3. Antibacterial Effects of NGW

Three NGW formulations were diluted with purified water to various concentrations (1%, 10%, and 50%), and their antibacterial activity was investigated. Based on findings from previous studies, an 8 h exposure time was used to determine the maximum effect.

**Figure 5:**
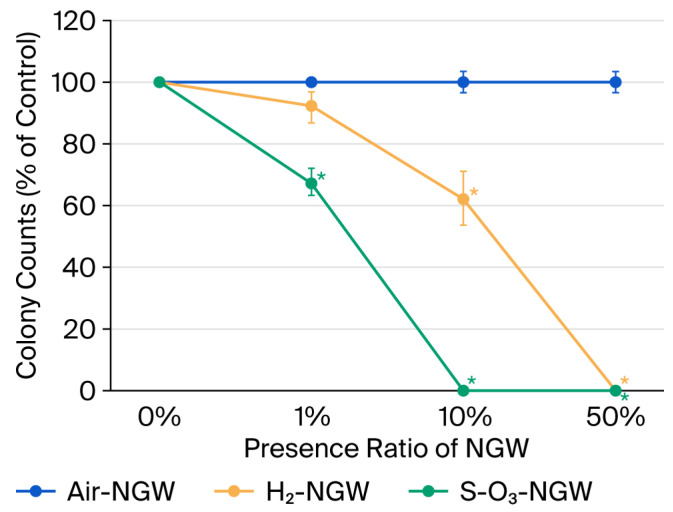
Antimicrobial effect of ultra-nano–pico-bubble water (NanoGAS water/NGW) on total viable bacteria (concentration dependence after 8 h of treatment). The colony count in the untreated control group was 302 ± 19 counts/dish. *: *p* < 0.001 vs. Air-NGW.

Generally, air-NGW exhibited negligible antibacterial activity; however, H_2_-NGW reduced the number of colonies by approximately 60% at 10% dilution and completely suppressed colony growth at 50% dilution. Moreover, O_3_-NGW exhibited more potent effects, demonstrating efficacy even at 1% dilution, whereas 100% suppression was achieved using 10% dilution (Figure 5).

**Figure 6:**
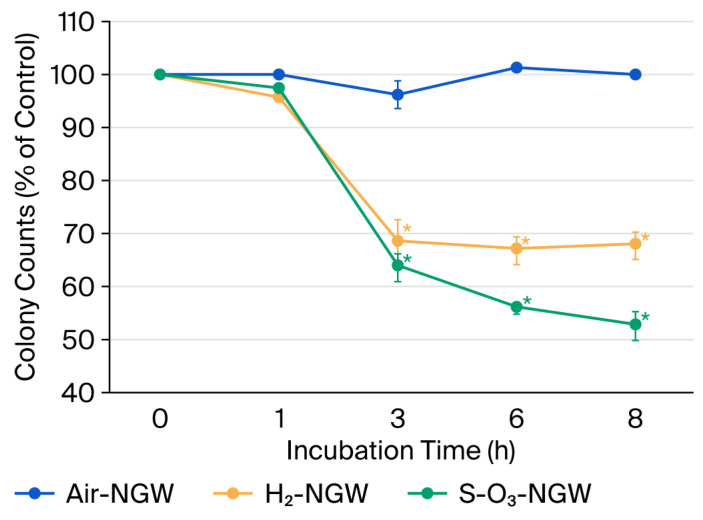
Antimicrobial effect of 1% ultra-nano–pico-bubble water (NanoGAS water/NGW) against total viable bacteria (time of effect onset). The colony count in the untreated control group was 278 ± 21 counts/dish. *: *p* < 0.001 vs. Air-NGW.

Therefore, all NGW formulations were diluted to a 1% concentration, and their time-dependent antibacterial effects were investigated. Air-NGW showed almost no effect, whereas H_2_-NGW significantly suppressed colony formation after 3 h. O_3_-NGW similarly suppressed colony formation after 3 h; however, its effects were stronger than those of H_2_-NGW, demonstrating nearly 50% suppression after 8 h (Figure 6).

### 2.4. Additive Antibacterial Effects of NGW and VB

Given the pronounced activity of ozonated water, we subsequently prepared two formulations: one with conventional-sized bubbles (S-O_3_) and another with a larger bubble size (L-O_3_). We then investigated whether they demonstrate additive effects with VB2 and VB3, both of which possess antibacterial and antiviral effects.

**Figure 7:**
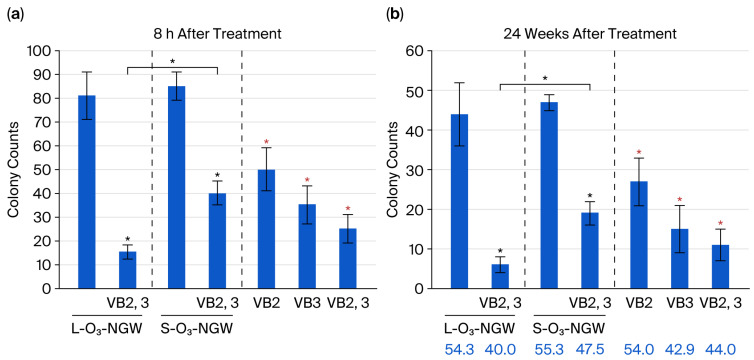
(**a**,**b**). Additive effect of ozonated ultra-nano–pico-bubble water (NanoGAS water/NGW); effect of O3-NGW and VB on total viable bacteria 8 h after treatment. L-O3-NGW; large-sized ozonated NanoGAS water, S-O3-NGW; small-sized ozonated NanoGAS water, VB2, 3; antiviral and bacterial active compound (polyoxometalate). *: *p* < 0.001 vs. L- or S-O3-NGW; *: *p* < 0.001 vs. untreated control. The colony count in the untreated control group was 316 ± 26 counts/dish. The numbers shown in blue are percentages of the colony count 8 h after treatment in (**a**).

Figure 7a shows the number of bacterial colonies following 8 h of exposure, with substantial bacterial inhibition observed for all formulations relative to the untreated control group (which yielded 320 colonies). For ozonated water alone, L-O_3_ reduced colony counts to 81 (19%), which was more pronounced compared to S-O_3_ (85; 20%); however, the difference was not statistically significant. The addition of VB2 and VB3 markedly enhanced the inhibitory effect, with S-O_3_ and L-O_3_ reducing colony counts to 40 and 15 (3.6%), respectively. This inhibitory effect exceeded that of VB alone as well as the combination of both VB2 and VB3. To evaluate the long-term stability of these effects, the samples were stored and re-examined after 24 weeks (Figure 7b).

The untreated control group yielded 316 colonies, showing no appreciable difference in colony count compared to that after 8 h. Moreover, compared to the control, the antibacterial activity of each treatment group was enhanced, exhibiting an approximately 50% increase in bacterial inhibition compared to that after 8 h.

**Figure 8:**
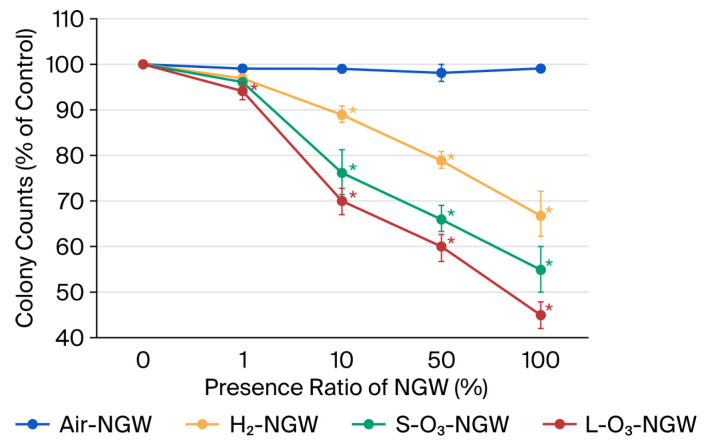
Additive antimicrobial effect of NGW on residual total bacteria in VB wet wipes. The colony count in the untreated control group was 98 ± 12 counts/dish (indicating concentration dependence after 8 h of treatment). *: *p* < 0.001 vs. Air-NGW.

Furthermore, the additive antibacterial effects of NGW against residual bacteria isolated from VB wet wipes were investigated. Figure 8 shows the colony inhibition effects when various NGWs were incubated with bacterial suspensions (VB-Bac) at different concentrations for 8 h. The initial number of colonies was 98, and all NGWs reduced colony counts in a concentration-dependent manner. Furthermore, a stronger additive effect was observed with O_3_-NGW than with H_2_-NGW, with L-O3 exhibiting more potent effects than S-O_3_.

## 3. Discussion

Currently, no strict standards exist for the production of NPB. Consequently, diverse products are currently available commercially, depending on the intended applications and production techniques of the manufacturer. Accordingly, efficacy claims cannot be made; however, the production of NPB with comprehensively characterized dissolved elements and well-defined bubble sizes may offer extensive benefits. We began producing NGW, which has finer bubbles than NPB, as a medical auxiliary solution around 2000 and have since expanded its applications as a solvent for bacterial solutions in FMT. Although physiological saline is typically used in FMT solvents, it has been reported that replacing saline with NGW increases the efficiency of bacterial engraftment in the intestinal epithelium. We have accumulated clinical evidence of its efficacy from over 700 patient cases with various conditions (irritable bowel syndrome, marine colitis, atopic dermatitis, allergies, constipation, etc.). Recently, clinical trials have been conducted in ASD patients, which have demonstrated favorable outcomes [56]. Although NGW has demonstrated promising potential applications, it remains underutilized in either medical or non-medical fields. Extensive research has been conducted to generate bubble water with long-term stability. We believe that stable NGW was successfully generated through the combination of a gas–liquid mixed fluid generator, a rotary mixer, and a bubble shear filter. In this system, negatively charged bubbles in the gas–liquid mixture are influenced by electromagnetic forces when moving through a static magnetic field, causing them to become static. When the static fluid is subsequently introduced into the rotary mixer, the initial velocity at introduction tends to be higher than that when it is introduced as a turbulent fluid, resulting in increased fluid acceleration and flow velocity as it flows through the spiral shape. The current detection limit for particle size is approximately 2 nm, with the analysis revealing that a substantial number of bubbles with diameters of 0.2 mm or less remained stable (Figure 1). Conversely, bubbles larger than 1 mm (microbubbles) disappeared owing to the self-pressurizing effect caused by buoyancy. It has been shown that the long-term stability of NGWs stems from Brownian motion, which helps maintain the balance between buoyancy and gravity.

Recent studies have reported difficulties in generating stable NGWs [48] due to the strong correlation between bubble size and lifespan [2], with greater efficacy observed against colitis and colorectal cancer with smaller bubble sizes [25]; we believe that this prolonged stability enables accurate, long-term evaluations of the various potential activities of NGW.

In this study, we focused on incorporating oxygen, hydrogen, and ozone into NGW owing to their high versatility. In particular, the aroma component, citral, used in our hand towels is highly volatile, and maintaining its fragrance for extended periods has represented a major challenge.

Citral exists as two isomers (neral and geranial), and similar spectra were obtained via mass spectrometry. However, there was no chromatographic interference, and clear separation was established, with retention times of approximately 10.3 and 10.7 min.

The calibration curve demonstrated good linearity, with a correlation coefficient of 0.9949, indicating that the citral in the NGW (combined peak areas of the two isomers) could be quantified.

Although citral exhibits poor solubility in water (0.59 mg/mL), several NGWs demonstrated enhanced solubility two weeks after preparation (Table 1a). However, after four weeks, their solubility decreased below this level, suggesting that long-term inclusion may not be achieved (Table 1b). Additionally, increasing the amount of citral added to O_3_-NGW (Sample #3) increased solubility but did not improve the yield. Therefore, further investigations of synergistic combinations with CD or alternative methods may be necessary to dramatically improve the inclusion effect.

In addition, because our manufactured products contained antibacterial and antiviral PM components (such as VB), we believe that developing formulations that exhibit additive effects will enable us to respond to future environmental challenges, such as emerging viruses.

Previous studies have already investigated the antibacterial effects of NGW on *E. coli* and *S. aureus*, indicating that the maximal effect was achieved following exposure for more than 6 h, with similar effects observed for H_2_-NGW and O_3_-NGW [58]. However, our evaluations against general viable bacteria revealed that O_3_-NGW demonstrated markedly stronger antibacterial effects than H_2_-NGW. Although H_2_-NGW has been suggested to exhibit antioxidant activity [12], O_3_-NGW is believed to kill bacteria through oxidation [15,16]. Therefore, their comparable antimicrobial effects remain a curious phenomenon warranting further investigation.

The bacterial colony counts after diluting NGW with purified water and allowing exposure for 8 h are shown in Figure 5. Both H_2_-NGW and O_3_-NGW exhibited 100% inhibition at a 50% dilution, with O_3_-NGW demonstrating stronger inhibition than H_2_-NGW at a 10% dilution, maintaining 100% suppression. Notably, even at a concentration of 1% (1/100 of the total amount), O_3_-NGW achieved approximately 70% inhibition.

Therefore, the effects of exposure time were subsequently investigated using 1% NGW (Figure 6). Although the bacterial colony count in the untreated control was lower (278 vs. 302 in Figure 5), a slightly stronger inhibitory effect was observed. Near-maximal inhibitory effects were achieved following 3 h or more of exposure, with O_3_-NGW consistently exhibiting stronger effects than the other NGW formulations. This observation that O_3_-NGW achieves these antibacterial effects even at a 100-fold dilution is expected to reduce costs.

Figure 7a,b present the antibacterial activity, expressed as colony counts, following 8 h and 24 weeks of combined treatment with O_3_-NGW or VB2 and VB3 (individually and in combination), which exhibit antibacterial and antiviral activity. The colony counts in the untreated control group remained largely unchanged even after 24 weeks of storage (8 h: 320 vs. 24 weeks: 316). Among the two 1% O_3_-NGW solutions, the large-sized bubble formulation demonstrated slightly stronger activity than the small-sized bubble formulation, although there was no significant difference. An additive effect was observed when VB2 and VB3 were added; however, L-O_3_-NGW exhibited a stronger additive effect than S-O_3_-NGW, indicating a marked difference between the two. The bubble densities were estimated to be 30 × 10^15^ bubbles/mL for S-O_3_-NGW and 10 × 10^15^ bubbles/mL for L-O_3_-NGW. Although L-O_3_-NGW had approximately one-third the number of bubbles, it is speculated that its superior efficacy may be because a larger amount of ozone molecules entered a single bubble or a substitution reaction occurred wherein VB molecules entered the bubbles, allowing ozone to act directly on the bacteria. However, further studies exploring combinations of bubble sizes and VB concentrations may help clarify their synergistic effects.

These samples were stored and re-examined after 24 weeks (Figure 7b) to assess long-term stability.

Compared to the untreated control, all groups exhibited enhanced inhibitory activity while maintaining an overall trend similar to the results obtained following 8 h of exposure. The inhibitory effect increased by approximately 50% compared to those observed after 8 h. Overall, these findings indicate that both NGW and VB retained their antibacterial effects over prolonged periods.

Furthermore, potential additive effects were investigated by treating residual bacterial suspension (VB-Bac) isolated from the VB-containing wet wipes with various dilutions of NGW. Notably, reproducible concentration-dependent additive antimicrobial effects were observed, and these additive effects were maintained even in long-term storage samples, suggesting that O_3_-NGW remained stable for extended periods. NGW bubbles encapsulate the bacterial cells, forming a dispersion in which the entire suspension exists as uniform colloidal particles; a bacteriostatic effect is achieved by inhibiting communication between the bacteria. Given the differing antibacterial mechanisms of NGW and VB, it is believed that each agent exerted its effect without inhibiting the other. While this paper reports the discovery of novel biological activity, future work must address the evaluation of NGW’s physicochemical properties and the mechanisms underlying the maintenance of its long-term activity. Furthermore, although translating these findings into practical applications requires additional study—including an assessment of cost-effectiveness—this work establishes a fundamental framework for future research.

## 4. Materials and Methods

### 4.1. Ultra-Nano–Pico-Bubble Water (NanoGAS Water/NGW)

The NGW was produced entirely at the Symbiosis in-house manufacturing plant (Osaka, Japan) according to the method specified in Patent No. 7028499, 2022-022321. Following production, the number of dissolved particles was measured using a Coulter counter (Multisizer 4e; Beckman Coulter Inc., Tokyo, Japan). This method maximizes the device’s measurement sensitivity by minimizing the time required for determining the initial particle velocity; the resulting data is then corrected based on the molecular weights of hydrogen or ozone and converted into a cell count. Because gas displacement is performed within a closed space using purified water that has passed through a reverse osmosis membrane, there is no background count, so contamination by impurities is effectively ruled out. Oxygen NanoGAS water, which was measured as a reference, shows a concentration of 400 × 10^15^ particles/mL; as the analysis is relative, no separate control is required. Four types of NGW were evaluated: Air-NGW, H_2_-NGW, S-O_3_-NGW, and L-O_3_-NGW.

The two types of ozonated water (S-O_3_ and L-O_3_) were classified based on the measured number of bubbles in the oxygenated water. Different bubble sizes can be obtained by adjusting the bubble shear time. S-O_3_ contained small-sized (3–10 nm) O_3_ bubbles, with a particle concentration of 30 × 10^15^ particles/mL, while L-O_3_ contained large-sized (7–20 nm) bubbles, with a particle concentration of 10 × 10^15^ particles/mL.

### 4.2. Citral

The citral used in this study was manufactured by Shandong Nhu Pharmaceutical Co., Ltd. (Weifang City, China) and purchased from Takacho Co., Ltd. (Tokyo, Japan).

### 4.3. Cyclodextrin

CD was purchased from FujiFilm Wako Pure Chemical Corporation (Osaka, Japan) and used as a positive control for inclusion experiments [67,68]. Preliminary studies confirmed that sufficient inclusion was achieved when the citral-to-cyclodextrin molar ratio was 50:1 (excess CD).

### 4.4. PM Compounds (VB)

VB2 (K_11_H[(VO)_3_(SbW_9_O_33_)_2_]·27H_2_O) and VB3 (Na_2_[SbW_9_O_34_]·19H_2_O) were synthesized at the FSX in-house synthesis laboratory according to the patented method described in International Patent Publication No. WO2019/230210. After purifying each raw powder via recrystallization, it was ground in a mortar, dissolved in ultrapure water, and filtered and sterilized using a 0.45 μm filter prior to use.

### 4.5. VB-Infused Wet Wipes

We employed VB-infused cloth wet wipes supplied and manufactured by FSX Corporation (Tokyo, Japan) according to their proprietary manufacturing method. The VB concentration in the wet wipes was confirmed to meet the product specifications by quantifying the metal elements (tungsten (W) and antimony (Sb)) in the compound using an energy-dispersive X-ray fluorescence analyzer (EDX-7000, Shimadzu Corporation, Kyoto, Japan) (Supplement Table S1).

### 4.6. Isolation and Preparation of the Standard Bacterial Suspension

All procedures were performed aseptically in a clean box within the laboratory.

A section of a standard cloth wet wipe was placed on a lysogeny broth (LB) agar medium (Kanto Chemical Co., Inc., Tokyo, Japan), and the resulting colonies were collected and cultured by shaking in a liquid LB medium (Kanto Chemical Co., Inc.) at 35 °C for 48 h. Serially diluted samples were spread onto Petri dishes using a CompactDry TC general viability test kit (FujiFilm Wako Pure Chemical Corporation, Osaka, Japan), and the bacterial concentration of the stock solution was calculated via colony counting (Std-Bac) and determined to be 5 × 10^6^ CFU/mL.

To prepare the VB bacterial suspension (VB-Bac), 10 mL/g of NGW was added to a VB-containing wet wipe, and the mixture was stirred in a vortex mixer for 60 s to extract the residual bacteria. The extract was centrifuged at 5000 rpm for 10 min, and the supernatant was collected and used as VB-Bac for subsequent experiments. All specimens treated with Std-Bac, VB-Bac, and NGW were stored in sealed amber glass vials at 23 ± 3 °C.

### 4.7. General Viability Test Kit

A CompactDry TC general viability test kit was purchased from FujiFilm Wako Pure Chemical Corporation (Osaka, Japan) and used according to the manufacturer’s instructions.

### 4.8. Evaluation of Citral Inclusion

To evaluate the effects of citral inclusion, 1 mL of NGW (air-NGW, H_2_-NGW, S-O_3_-NGW, or L-O_3_-NGW) or a 1% CD solution was mixed with 5 µL of the citral solution and stirred using a vortex mixer for 60 s.

The resulting solutions were then stored at room temperature (23 ± 3 °C) for 2 and 4 weeks prior to GC/MS analysis.

For GC/MS analysis, each sample was diluted 40-fold with methanol to obtain a solution containing approximately 250 ppm of citral. GC/MS was then performed using an Agilent 7890 GC System gas chromatograph coupled with an Agilent 5977B MSD mass spectrometer (Agilent Technologies, Inc., Tokyo, Japan). The measurement parameters used are listed in Table 2. Since citral exists as two isomers (neral and geranial), the total citral concentration was calculated as the sum of the individually quantified isomers.

### 4.9. Antibacterial Effect

Purified water or three types of NGW (H2-NGW, S-O3-NGW, and L-O3-NGW) (9.9 mL) were mixed with a viable bacterial suspension (0.1 mL) and incubated at 35 °C. After a predetermined time, a 1 mL sample was collected and added to the Petri dish of a general viable bacteria test kit; following incubation at 30 °C, the number of colonies that appeared was counted.

### 4.10. Statistical Analysis

All experiments were conducted using three samples, and the results are presented as the mean ± standard deviation. Significant differences between groups were determined using Student’s *t*-test, with *p* < 0.05 considered statistically significant. For qRT-PCR, a difference of 2 or more from the ΔΔCt value (a difference of 4 times or more at the mRNA level) was considered statistically significant.

## 5. Conclusions

The successful development of NPB, characterized by exceptionally small bubble sizes, enabled the production of bubble water with exceptional long-term stability. These NGW formulations demonstrated a slight capacity to dissolve aromatic components and citral compared to ordinary purified water. Furthermore, they exhibited marked antibacterial activity against general bacteria, with O_3_-NGW notably remaining effective even at 1% dilution and maintaining its activity for up to 24 weeks. Furthermore, an additive effect was observed when NGW was used in combination with VB—another type of antimicrobial substance—suggesting its high versatility and broad range of applications.

## Figures and Tables

**Table 1:** (**a**,**b**) Citral concentration in the sample (sum of neral and geranial values).

Sample	Area Value		Combined Area Value	Concentration (mg/mL)	Yield (%)
	Neral	Geranial			
(**a**) Measurements taken 2 weeks after inclusion ^a^					
Air-NGW	165,660.63	247,409.43	413,070.06	0.67	13.4
H_2_-NGW	274,927.88	268,865.84	543,793.72	0.83	16.6
S-O_3_-NGW	303,337.42	307,951.31	611,288.73	0.92	18.4
CD-pure water	1,360,249.78	3,074,507.4	4,434,757.18	4.21	84.2
(**b**) Measurements taken 4 weeks after inclusion ^b^					
S-03-NGW#1	415,038.70	501,781.92	916,820.62	0.27	5.4
S-03-NGW#2	459,789.87	505,594.66	1,279,242.34	0.28	5.6
S-03-NGW#3	564,894.72	714,347.62	965,384.53	0.35	4.7
L-03-NGW#1	467,087.30	522,875.64	989,962.94	0.28	5.6
L-03-NGW#2	547,635.38	595,168.35	1,142,803.73	0.32	6.4
L-03-NGW#3	731,608.47	970,262.64	1,701,871.11	0.44	5.9

^a^ Retention time: neral: 10.368 min and geranial: 10.77 min. ^b^ Retention time: neral: 10.35 min and geranial: 10.75 min. S-03-NGW#3 and L-03-NGW#3 were treated with 1.5 times the amount of citral.

**Table 2:** GC/MS measurement conditions.

GC	
System	7890B GC System
Inlet	Temp. 300 °C, Constant flow
Column	HP-5 Ultra Inert, 30 m, 0.25 mm ID, 0.25 μm (Film thickness)
Gas	He, 2 mL/min
Oven	50 °C (1 min)—10 °C/min—200 °C—5 °C/min—300 °C (4 min)
Injection	1 mL, Split (5:1)
Trans line	320 °C
MS	
System	5977B MSD (Inert Plus)
Ion source	EI (280 °C)
Range	*m*/*z* 40–550
Scan	2.9 scan/s

## Data Availability

The original contributions presented in this study are included in the article. Further inquiries can be directed to the corresponding author.

## Supplementary Materials

The following supporting information can be downloaded at: https://www.mdpi.com/article/10.3390/ijms27156909/s1.

## Abbreviations

The following abbreviations are used in this manuscript:

ASD	Autism spectrum disorder
CD	Cyclodextrin
FMT	Fecal microbiota transplantation
NPB	Nano–pico-bubble water
NGW	NanoGAS water/ultra-nano–pico-bubble water
PMs	Polyoxometalates
rCDI	Recurrent Clostridium difficile infection
TIC	Total ion chromatography
VB	VirusBlock
VB-Bac	VB bacterial suspension
