# Supplementary material for: Generation and Application of Ultra-Fine, Long-Term Stable Nanobubble Water: An Evaluation of Inclusion Effects on Aromatic Components and Antimicrobial Activity
Source: Int J Mol Sci. 2026 Aug 1;27(15):6909. doi: 10.3390/ijms27156909 (PMC13467179; doi:10.3390/ijms27156909)
Supplement: Supplementary file 1 [file ijms-27-06909-s001.zip › ijms-4424719-supplementary.pdf]

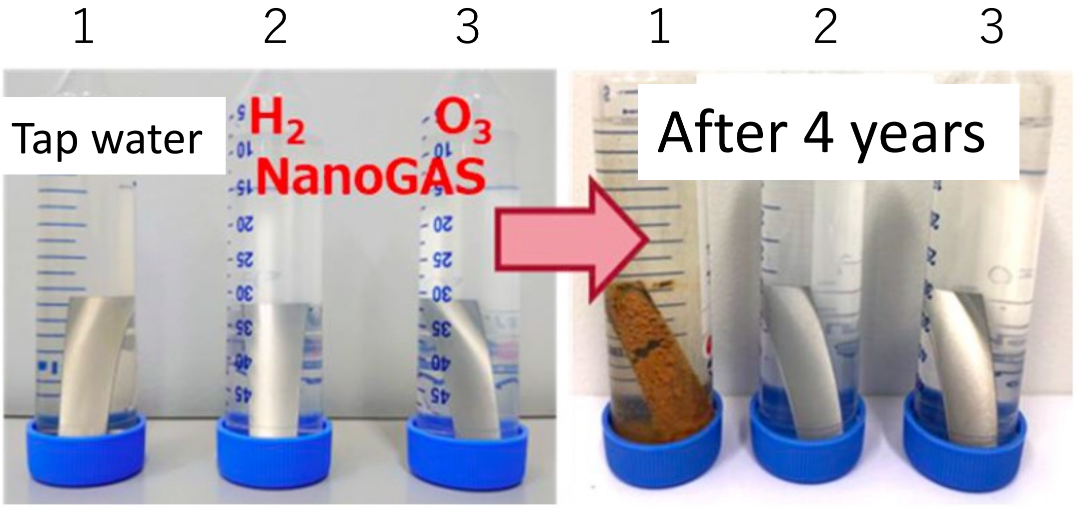

Figure S1. Rust prevention effect on aluminum pieces.

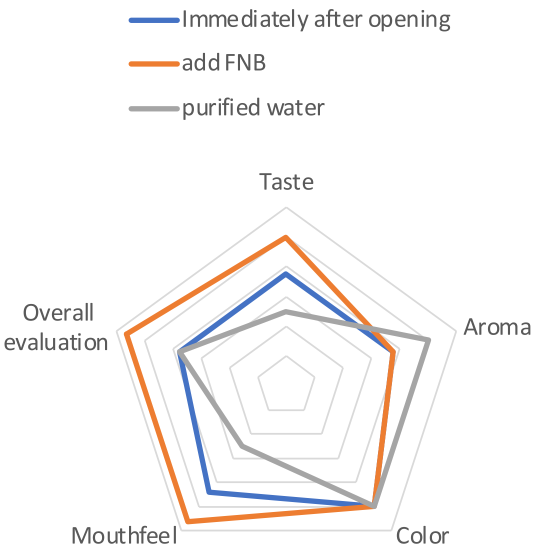

Hakushika Classic Unpasteurized Sake

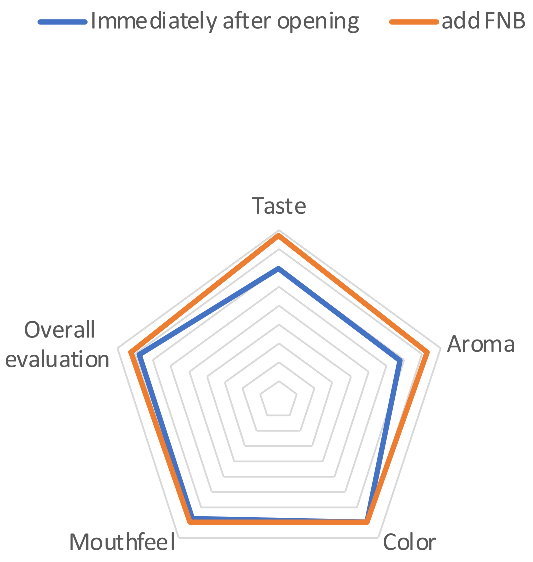

Kuromatsu Hakushika Unpasteurized Sake

	Immediately after	add FNB	purified water
Taste	3.750	4.000	3.500
Aroma	3.750	3.750	4.000
Color	4.000	4.000	4.000
Mouthfeel	3.875	4.125	3.500
Overall evaluation	3.750	4.125	3.750

	Immediately after	add FNB
Taste	3.500	4.375
Aroma	3.375	4.125
Color	4.000	4.000
Mouthfeel	3.875	4.000
Overall evaluation	3.875	4.125

Figure S2. Antioxidant effect on sake

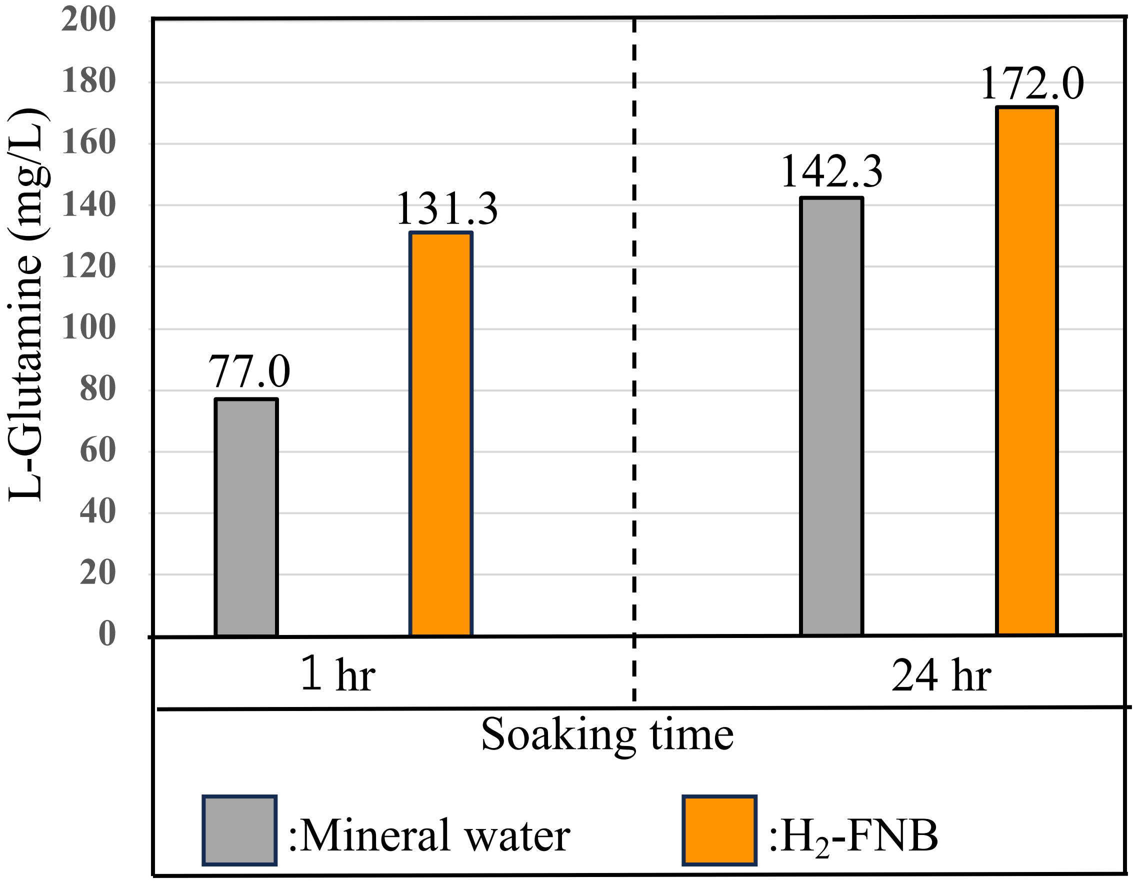

Figure S3. Verification of the extraction efficiency of umami components using cold-brewed kelp

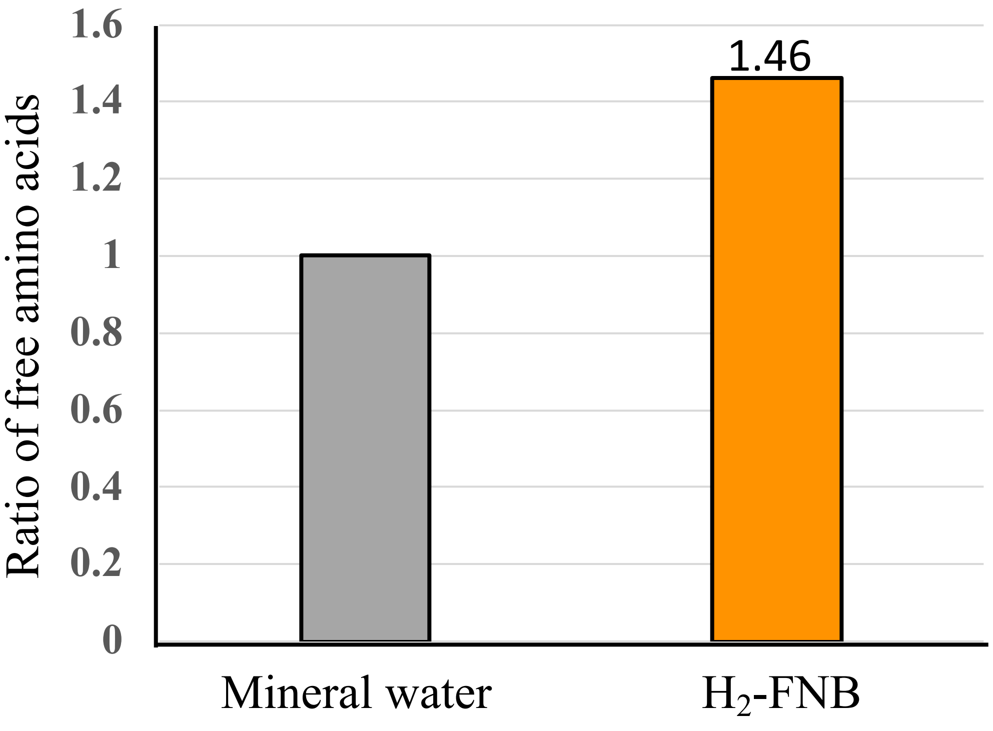

Figure S4. Fermentation-promoting effect of sweet koji
（Quantification of free amino acids by HPLC）

	1	2	3	mean	SD
W ug/l	246.06	205.03	265.75	238.95	30.98
Sb ug/l	118.79	165.47	85.34	123.20	40.25

Table S1. Measurement of VB content by elemental analysis using an energy-dispersive X-ray fluorescence spectrometer
